# Serum uric acid/creatinine ratio and free androgen index are synergistically associated with increased risk of polycystic ovary syndrome in obese women

**DOI:** 10.1186/s12902-022-01240-y

**Published:** 2022-12-13

**Authors:** Mervat M El-Eshmawy, Asmaa Ibrahim, Rania Bahriz, Nermeen Shams-Eldin, Nancy Mahsoub

**Affiliations:** 1 Internal Medicine Department, Mansoura Specialized Medical Hospital, Faculty of Medicine, Mansoura University, P.O. Box: 35516 Mansoura, Egypt; 2 Internal Medicine Department, Meet Salsil Central Hospital, Ministry of Health and Population, Meet Salsil, Egypt; 3 Gynecology and Obstetric Department, Faculty of Medicine, Mansoura University, Mansoura, Egypt; 4 Clinical Pathology Department, Faculty of Medicine, Mansoura University, Mansoura, Egypt

**Keywords:** Polycystic ovary syndrome, Serum uric acid/creatinine ratio, Free androgen index

## Abstract

**Background:**

Features of metabolic syndrome such as abdominal obesity, insulin resistance, hypertension and dyslipidemia are commonly encountered in polycystic ovary syndrome (PCOS). Recent evidence has suggested an association between high serum uric acid/creatinine **(**UA/Cr) ratio and metabolic syndrome however, no studies have investigated this association in PCOS. The current study was conducted to investigate the relationship between UA/Cr ratio and PCOS and to identify whether UA/Cr ratio and free androgen index (FAI) have an additive interaction for detection of PCOS risk in obese women.

**Methods:**

This study enrolled 40 obese women with PCOS and 40 control women with regular menstrual cycles matched for age and body mass index (BMI). Anthropometric measurements, fasting glucose, fasting insulin, homeostasis model assessment of insulin resistance (HOMA-IR), lipids profile, luteinizing hormone (LH), follicle stimulating hormone (FSH), estradiol, dehydroepiandrosterone sulfate (DHEAS), sex hormone binding globulin (SHBG), total testosterone, free androgen index (FAI), UA/Cr ratio were assessed.

**Results:**

Serum UA/Cr ratio was significantly higher in obese women with PCOS than in non-PCOS women. UA/Cr ratio was correlated with BMI, waist and neck circumferences, blood pressure, fasting insulin, HOMA-IR, lipids, LH/FSH, estradiol, DHEAS, total testosterone, FAI and SHBG. UA/Cr ratio and FAI were independent risk factors for PCOS in obese women however, the addictive interaction between UA/Cr ratio and FAI had a higher fold risk (OR: 4.3, 95% CI, 3.4–7.58) and a more significance (*P* = 0.002) for determination of PCOS.

**Conclusion:**

Serum UA/Cr ratio combined with FAI can exert an additive or synergistic impact on prediction of PCOS in obese women.

## Background

Polycystic ovary syndrome (PCOS) is the most common endocrine disorder in women of reproductive age [1] affecting 5–20% [2]. It is a multi-factorial complex disorder with genetic and environmental background [3]. PCOS is characterized by hyperandrogenism, chronic anovulation and polycystic ovaries after exclusion of related disorders [4]. Beyond infertility, women with PCOS are prone to many complications such as increased risk of type 2 diabetes (T2D), cardiovascular disease, sleep apnea syndrome, endometrial cancer in addition to mood disorders [5, 6] therefore, early diagnosis and treatment is of a great importance.

Hyperandrogenemia is one of the most important features of PCOS and the key point in its diagnosis [7]. Total testosterone level in women may not be a sensitive marker for the detection of androgen excess on the other side, routine measurement of free testosterone is challenging [8, 9]. Accordingly, free androgen index (FAI), calculated by total testosterone and sex hormone binding globulin (SHBG), indirectly reflects the free androgen levels [10]. FAI is considered to be the single most-useful test of hyperandrogenemia in women [11].

Women with PCOS frequently exhibit features of metabolic syndrome (MS) such as abdominal obesity, insulin resistance, hypertension and dyslipidemia [1, 12, 13]. Besides that, MS is highly prevalent in women with PCOS [14]. Recently, an increasing evidence indicates that elevated serum uric acid/creatinine (UA/Cr) ratio, a new biomarker reflecting endogenous uric acid levels more precisely than serum uric acid, is related to MS [15–18]. PCOS is a complex endocrine-metabolic disorder in which serum uric acid is closely related to androgen excess [19]. It is unknown whether the obese women with PCOS have an elevated UA/Cr ratio, and if so, whether the link between UA/Cr ratio and FAI provides a benefit to predict PCOS in obese women. This study was conducted to explore the relationship between UA/Cr ratio and PCOS and to identify whether UA/Cr ratio and FAI have an additive interaction for detection of PCOS risk in obese women.

## Methods

This case control study comprised 40 obese women with PCOS and 40 control women matched for age and body mass index (BMI). Obesity was defined as a BMI ≥ 30 Kg/m^2^. Healthy controls had regular menstrual cycles and no family history of PCOS. Women with PCOS were recruited from the Fertility Outpatient Clinic at Mansoura University Hospital, Mansoura University, Egypt.

All participants were subjected to a thorough medical history and underwent a clinical examination with stress on signs of hyperandrogenism (acne, hirsutism, and/or acanthosis nigricans). Systolic and diastolic blood pressure (SBP & DBP) were taken in the sitting position after 10 min of rest using a random-zero sphygmomanometer. Anthropometric measurements including height, body weight, BMI and waist circumference (WC) were obtained with standardized techniques. BMI was calculated as weight/height^2^ [kg/m^2^] and WC was measured at the level of the iliac crest at the end of normal expiration. Neck circumference, as an indicator for upper-body subcutaneous fat, was measured at the level of the thyroid cartilage in the standing position with erect head.

Diagnosis of PCOS was based on the Rotterdam revised criteria [4]; at least 2 of the 3 following criteria after exclusion of other causes of hyperandrogenism and menstrual irregularities:

1. Oligomenorrhea (intermenstrual interval of > 35 days and < 8 menstrual bleeds in a year) or amenorrhea (absent menstrual bleeding in the past 90 days).
2. Clinical hyperandrogenism (Ferriman Gallwey score ≥ 8 [20] and/or biochemical hyperandrogenism (increased total testosterone levels or dehydroepiandrosterone sulfate (DHEAS).
3. Polycystic ovaries on ultrasound scan (at least one ovary contains 12 follicles or more with 2 to 9 mm in diameters and/or ovarian volume > 10 mm).

In eumenorrheic women with clinical signs of hyperandrogenism, normal ovulation was assessed by a progesterone level on days 20–24 of 1 or 2 consecutive cycles.

None of the participants had thyroid disorders, hyperprolactinemia, non-classical congenital adrenal hyperplasia, T2D, hepatic or renal failure, connective tissue disorders, malignancy, pregnancy, taking birth control pills or hormone replacement therapy, anti-androgens, ovulation-inducing agents, steroids, lipid lowering drugs or insulin sensitizers for at least 3 months before the study. We also excluded women who were taking medications affecting the circulating uric acid levels such as aspirin and thiazide diuretics.

### Laboratory assay

Fasting plasma glucose, serum uric acid, creatinine, total cholesterol (TC), triglycerides (TGs) and high density lipoprotein cholesterol (HDL-C) were measured by automated chemistry analyzer (cobas c311) using its commercial kits supplied by Roche Diagnostic Germany. Low density lipoprotein cholesterol (LDL-C) was calculated according to Friedewald formula [21]. Fasting insulin was estimated by a solid phase enzyme-linked immunosorbent assay supplied by (BIOS) kits. Homeostasis model assessment of insulin resistance (HOMA-IR) was calculated with the formula: HOMA-IR= [fasting insulin (µU/mL) × fasting glucose (mmol/L)/22.5] [22]. Serum luteinizing hormone (LH), follicle stimulating hormone (FSH), estradiol, testosterone, DHEAS and SHBG were measured by automated chemistry analyzer (Cobas 411) using a potential electrochemiluminescence technology for immune assay analysis. Serum UA/Cr ratio was calculated as uric acid (mmol/l)/creatinine (µmol/l). FAI was calculated with the formula: total testosterone (nmol/L)/SHBG (nmol/L) ×100 [10].

### Statistical analysis

Data entry and analysis were done by the SPSS statistical package (version 22, Armonk, NY: IBM Corp). The data were expressed as mean ± SD for continuous data, number and percent for categorical data and median (minimum-maximum) for skewed data. Student´s t and Mann-Whitney U tests were used to compare the 2 studied groups for parametric and non-parametric data, respectively. The correlations of UA/Cr ratio and FAI with all other studied variables were analyzed by the Pearson and Spearman correlations analysis. Binary stepwise logistic regression analysis was performed to predict the independent variables of binary outcome; significant predictors in the univariate analysis were entered into regression model. *P* ≤ 0.05 was considered to be significant.

## Results

**Fig. 1:**
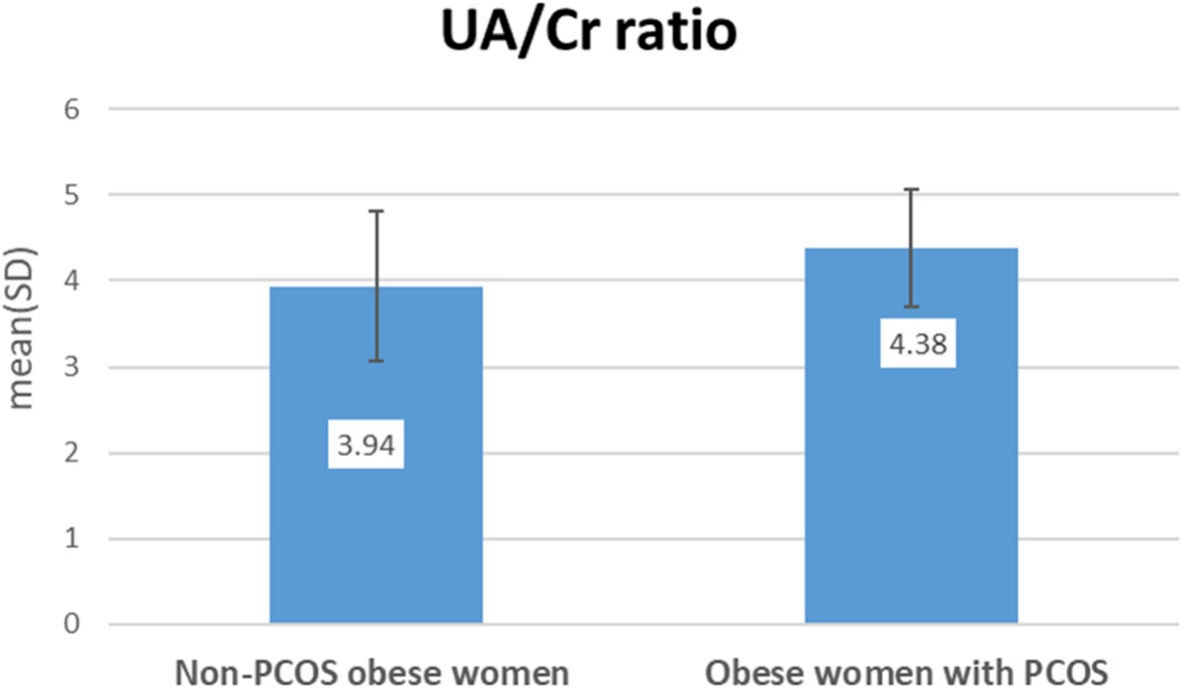
UA/Cr ratio in obese women with and without PCOS

Obese women with PCOS had significantly higher SBP, DBP, FPG, fasting insulin, HOMA-IR, LH/FSH, DHEAS, total testosterone, FAI, uric acid and lower SHBG compared with non-PCOS obese women. No significant differences between obese women with and without PCOS with regard to TC, TGs, LDL, HDL, estradiol and creatinine Table 1. UA/Cr ratio was significantly higher in obese women with PCOS than in non-PCOS women (4.38 ± 0.69 vs. 3.94 ± 0.88, *P* < 0.001) Fig. 1.

**Table 1:** Baseline characteristics of the study subjects

Variables	Obese women with PCOS (*n* = 40)	Non-PCOS obese women (*n* = 40)	*P*
**Age (years)**	28.68 ± 5.65	26.98 ± 6.67	0.225
**BMI (Kg/m** ^**2**^ **)**	34.77 ± 4.95	33.85 ± 4.09	0.367
**WC (cm)**	102.06 ± 10.09	98.93 ± 7.68	0.122
**Neck circumference (cm)**	37.30 ± 1.99	36.88 ± 1.86	0.326
**SBP (mm Hg)**	125.18 ± 6.74	119.0 ± 3.62	< 0.001*
**DBP (mm Hg)**	81.70 ± 2.76	78.88 ± 3.49	< 0.001*
**FPG (mmol/l)**	4.67 ± 0.53	4.34 ± 0.53	0.006*
**Fasting insulin** (**µU/ml)**	4.77 (3.8–5.4)	4.38 (3.5–5.22)	< 0.001*
**HOMA-IR**	3.6 (0.7–26)	1.5 (0.6–4.10)	< 0.001*
**TC (mmol/l)**	4.13 (1.71–6.72)	3.67 (1.89–5.64)	0.098
**TGs (mmol/l)**	1.21 (0.66–3.39)	1.23 (0.76–2.60)	0.541
**LDL-C (mmol/l)**	2.10 (0.91–4.66)	2.165 (1.17–4.53)	0.941
**HDL-C (mmol/l)**	1.235 (0.36–2.5)	1.15 (0.39–2.51)	0.967
**LH/FSH** (**IU/L)**	3.77 (1.03–5.38)	1.11 (1.0-1.22)	< 0.001*
**Estradiol (pmol/L)**	235.69 (80.57–396.5)	201.31 (97–311)	0.064
**DHEAS (µmol/L)**	6.37 (2.18–9.98)	3.93 (1.9–7.78)	< 0.001*
**SHBG (nmol/L)**	33.05 (23.1–70.3)	78.15 (38.6–128)	< 0.001*
**Total testosterone (nmol/L)**	2.58 (0.77–4.2)	0.98 (0.09–2.42)	< 0.001*
**FAI**	8.75 (1.55–11.55)	1.21 (0.13–3.43)	< 0.001*
**Uric acid (µmol/l)**	343.86 ± 54.75	289.25 ± 44.39	< 0.001*
**Creatinine (µmol/l)**	79.32 ± 11.58	75.58 ± 14.01	0.197
**UA/Cr ratio**	4.38 ± 0.69	3.94 ± 0.88	< 0.001*

Data are expressed as mean ± standard deviation, or median (minimum-maximum), *PCOS* Polycystic ovary syndrome, *BMI* Body mass index, *WC* Waist circumference, *SBP* Systolic blood pressure, *DBP* Diastolic blood pressure, *FPG* Fasting plasma glucose, *HOMA-IR* Homeostasis model assessment of insulin resistance, *TC* Total cholesterol, *TGs* Triglycerides, *LDL-C* Low density lipoprotein cholesterol *HDL-C* High density lipoprotein cholesterol, *LH* Luteinizing hormone, *FSH* Follicle stimulating hormone, *DHEAS* Dehydroepiandrosterone sulphate, *SHBG* Sex hormone binding globulin, *FAI* Free androgen index, *UA/Cr* Uric acid/creatinine.

*P is significant if ≤ 0.05

**Fig. 2:**
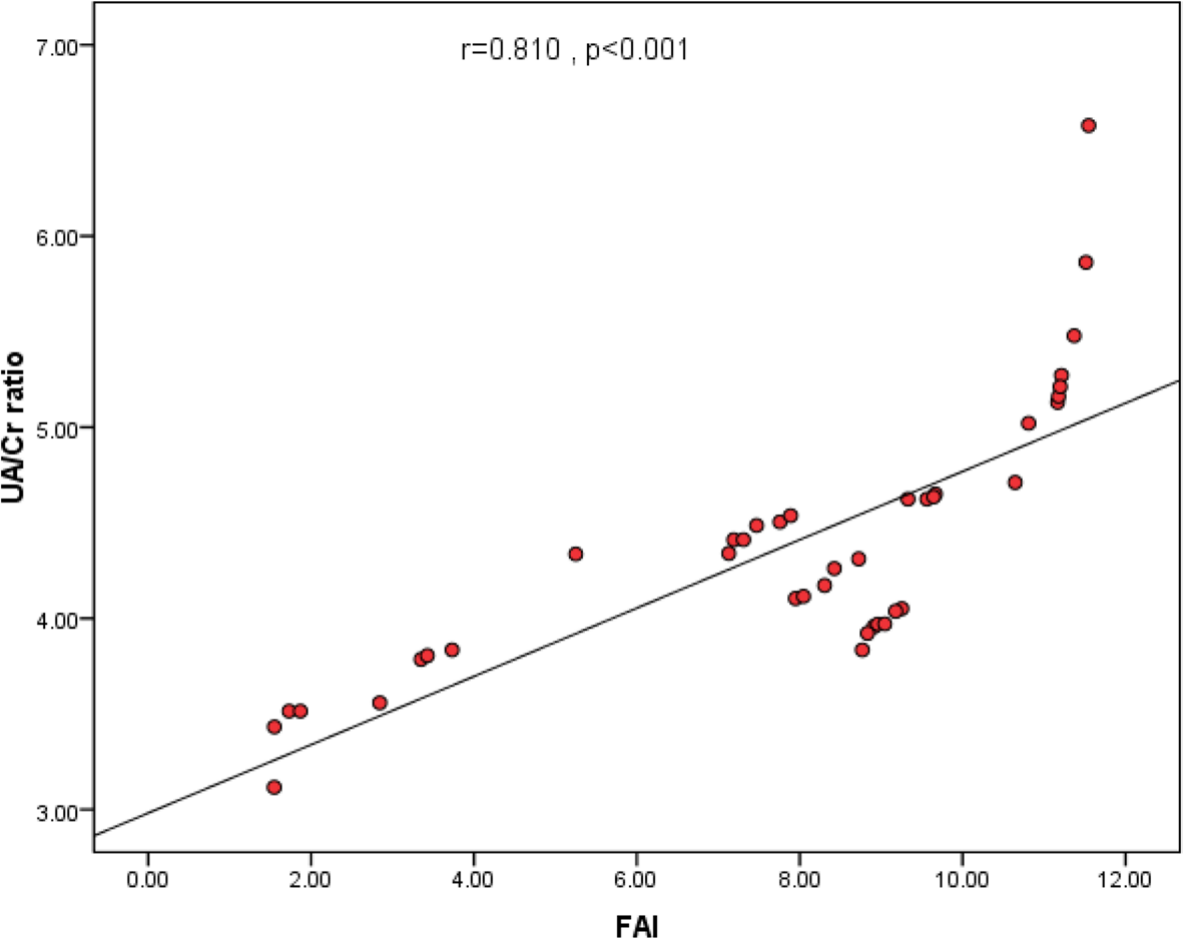
Correlation between UA/Cr ratio and FAI in obese women with PCOS

UA/Cr ratio was positively correlated with BMI, WC, neck circumference, SBP, DBP, fasting insulin, HOMA-IR, TC, TGs, LDL-C, LH/FSH, estradiol, DHEAS, total testosterone and FAI (Fig. 2) and negatively correlated with HDL-C and SHBG. FAI was positively correlated with BMI, WC, neck circumference, fasting insulin, HOMA-IR, TC, TGs, LDL-C, LH/FSH, estradiol and DHEAS and negatively correlated with HDL-C Table 2.

**Table 2:** Correlations between UA/Cr ratio and FAI with other variables in obese women with PCOS

Variables	UA/Cr ratio		FAI	
	*r*	*P*	*r*	*P*
**Age (years)**	0.157	0.334	0.142	0.382
**BMI (Kg/m** ^**2**^ **)**	0.499	0.001*	0.511	0.001*
**WC (cm)**	0.998	0.001*	0.816	< 0.001*
**Neck circumference (cm)**	0.539	0.001*	0.333	0.036*
**SBP (mm Hg)**	0.319	0.045*	0.083	0.609
**DBP (mm Hg)**	0.658	< 0.001*	0.026	0.875
**FPG (mmol/l)**	0.073	0.655	0.06	0.715
**Fasting insulin** (**µU/ml)**	0.531	< 0.001*	0.398	0.01*
**HOMA-IR**	0.312	0.05*	0.415	0.04*
**TC (mmol/l)**	0.729	< 0.001*	0.711	< 0.001*
**TGs (mmol/l)**	0.496	0.001*	0.517	< 0.001*
**LDL-C (mmol/l)**	0.727	< 0.001*	0.538	< 0.001*
**HDL-C (mmol/l)**	-0.839	< 0.001*	-0.691	< 0.001*
**LH/FSH** (**IU/L)**	0.618	< 0.001*	0.465	0.002*
**Estradiol (pmol/L)**	0.940	< 0.001*	0.689	< 0.001*
**DHEAS (µmol/L)**	0.453	0.003*	0.415	0.008*
**SHBG (nmol/L)**	-0.572	< 0.001*	-	-
**Total testosterone (nmol/L)**	0.733	< 0.001*	-	-
**FAI**	0.810	< 0.001*	-	-

*PCOS* Polycystic ovary syndrome, *BMI* Body mass index, *WC* Waist circumference, *SBP* Systolic blood pressure, *DBP* Diastolic blood pressure, *FPG* Fasting plasma glucose, *HOMA-IR* Homeostasis model assessment of insulin resistance, *TC* Total cholesterol, *TGs* Triglycerides, *LDL-C* Low density lipoprotein cholesterol, *HDL-C* High density lipoprotein cholesterol, *LH* Luteinizing hormone, *FSH* Follicle stimulating hormone, *DHEAS* Dehydroepiandrosterone sulphate, *SHBG* Sex hormone binding globulin, *UA/Cr* Uric acid/creatinine, *FAI* Free androgen index.

*P is significant if ≤ 0.05

After adjustments for confounding factors, the UA/Cr ratio and FAI were independent risk factors for PCOS in obese women; the OR (95% CI) were 1.62 (1.13–4.58) and 3.12 (1.28–8.25), respectively. Furthermore, the addictive interaction between UA/Cr ratio and FAI was an independent determinant for PCOS with a higher fold risk (OR: 4.3, 95% CI: 3.4–7.58) and a more significance (*P* = 0.002) Table 3.

**Table 3:** Associations of UA/Cr ratio, FAI and combined UA/Cr ratio & FAI with PCOS

Variables	Crude model		Adjusted model	
	**OR (95% CI)**	*P*	**OR (95% CI)**	*P*
**UA/Cr ratio**	2.06 (1.12–3.79)	0.02*	1.62 (1.13–4.58)	0.03*
**FAI**	3.72 (1.63–8.56)	0.002*	3.12 (1.28–8.25)	0.003*
**UA/Cr ratio + FAI**	4.8 (1.01–6.78)	< 0.001*	4.3 (3.4–7.58)	0.002*

*OR* Odds ratio, *CI* Confidence interval, *UA/Cr U*ric acid/creatinine, *FA* Free androgen index, *PCOS* Polycystic ovary syndrome.

*P is significant if ≤ 0.05

## Discussion

In the current study, obese women with PCOS had a significantly higher UA/Cr ratio than those without PCOS. UA/Cr ratio was significantly correlated with BMI, WC, neck circumference, blood pressure, fasting insulin, HOMA-IR and lipids profile. Additionally, UA/Cr ratio was associated with PCOS risk in obese women after adjusting for confounding factors.

Our results are in accordance with Al-Daghri et al. [15] who noticed significant correlations between serum UA/Cr ratio and BMI, WC and HDL-C in patients with T2D. Our findings are also in parallel with Moriyama [16], who found significant associations between increased UA/Cr quartiles and anthropometric measures, blood pressure, insulin resistance, lipids and number of MS components in healthy Japanese subjects. UA/Cr ratio was positively correlated with BMI, WC, TG, LDL-C, HOMA-IR, and negatively correlated with HDL-C even in subjects with normal serum uric acid levels and different glucose tolerance states [23]. We found a positive correlation between UA/Cr ratio and neck circumference, this is in parallel with Yang et al. [24] who identified a positive correlation between neck circumference and hyperuricemia in women with PCOS. Upper body adiposity leads to excess free fatty acid release and high airway pressure with consequent oxidative stress and insulin resistance [25].

The close association between elevated serum uric acid, the end product of purine metabolism, and PCOS has been previously reported [19, 26] on the other hand, obesity is thought to be the driver of renal disease in women with PCOS [27]. Uric acid and creatinine levels are positively associated with the most metabolic disorders related to PCOS such as obesity, dyslipidemia and hypertension [28–32]. Additionally, a relationship between serum creatinine and hyperuricemia in both men and women has been previously established [30, 33] thus, the application of the UA/Cr ratio reduces the interference due to sex and renal function [34]. Indeed, serum UA/Cr ratio reflects the endogenous uric acid levels more precisely than uric acid and is closely related to metabolic disorders [35]. The association of UA/Cr ratio with MS and its components has been recently proposed [15–18]; the UA/Cr may be a useful marker in the pathogenesis and prognosis of MS [15] and a good indicator for components of MS even in healthy subjects [16]. MS and PCOS are almost the two sides of the same coin thus, the association between high UA/Cr ratio and PCOS in obese women is an expected finding.

In the present study, FAI was significantly higher in obese women with PCOS than in those without PCOS. FAI was significantly correlated with most of PCOS worse metabolic profiles. FAI was an independent risk factors for PCOS in obese women with adjusted OR 3.12.

Our findings are in agreement with the previous reports investigating the relationship between FAI and PCOS [36–39]. We found a significant correlation between FAI and metabolic disorders related to PCOS. In an earlier study conducted by Kauffman et al. [40], FAI was correlated with fasting insulin, HOMA-IR, TC, LDL in PCOS women with NFLD. Our results also go in line with Cai et al. [41] who observed that FAI was positively associated with WC, BMI, TG, TC and LDL-C and negatively associated with HDL-C. Recently, de Medeiros et al. [42] reported a positive association of FAI with most anthropometric and metabolic biomarkers in PCOS women. Al Kindi et al. [43] recommended the use of FAI than testosterone alone in the routine investigation for hyperandrogenism in women. Despite FAI was speculated to be more accurate than total testosterone to evaluate androgen excess in women with PCOS [44, 45], a recent meta-analysis revealed that FAI has a moderate diagnostic value for PCOS [46]. Of interest, we observed a significant positive association between UA/Cr ratio and FAI. Our study also revealed a new combined index composed of FAI and UA/Cr ratio; this combination was an independent significant risk factor for PCOS in obese women with a 4.3-fold risk.

Serum uric acid is closely related to androgen excess which is the clue criterion of PCOS [19]: hyperandrogenemia increases serum uric acid by inducing hepatic metabolism of purine nucleotides and enhancing purine renewal in the kidney [47, 48]. Indeed, hyperandrogenemia is associated with visceral obesity, insulin resistance and low inflammatory state [49–51]. Accumulation of visceral adipose tissue stimulates uric acid synthesis through de novo purine synthesis in the pentose phosphate pathway [52] in turn, uric acid induces intracellular and mitochondrial oxidative stress leading to increased fat synthesis and impaired fatty acid oxidation through disturbances in the Krebs cycle [53, 54]. Furthermore, hyperinsulinemia reduces renal uric acid clearance by activation of GLUT 9 leading to hyperuricemia [55]. On the other hand, hyperuricemia induces insulin resistance through suppression of both basal and glucose-stimulated insulin secretion [56], induction of β-cell dysfunction through NF-κB signaling pathway [57], reduction of serum nitric oxide levels [58] and bioavailability [59].

To our knowledge, this is the first study to indicate the association of UA/Cr ratio with PCOS. In addition to the direct individual association of UA/Cr ratio and FAI with PCOS risk in obese women, we found that the combined UA/Cr ratio and FAI had an additive interaction for the detection of PCOS.

## Conclusion

Serum UA/Cr ratio combined with FAI can exert an additive or synergistic impact on prediction of PCOS in obese women. However, large-scale prospective studies of different ethnicities are needed to confirm our results.

## Data Availability

All data generated or analyzed during this study are included in this published article.
